# Lidocaine-Associated CNS Toxicity at Therapeutic Dosage: A Case Report and Literature Review

**DOI:** 10.7759/cureus.62231

**Published:** 2024-06-12

**Authors:** Rachel D Martin, Matthew Scanlon, Kerry McCabe

**Affiliations:** 1 Emergency Medicine, Charleston Area Medical Center, Charleston, USA; 2 Medical Toxicology, University of Pittsburgh Medical Center, Pittsburgh, USA

**Keywords:** neurotoxic, local anesthetic, local anesthetic systemic toxicity (last), medial branch nerve block, lipid emulsion, lidocaine toxicity

## Abstract

Lidocaine is a commonly used anesthetic. High doses or intravenous administration of lidocaine, as well as other local anesthetics, may result in systemic effects involving the cardiovascular and neurologic systems. Typically, effects are dependent on the serum concentrations of the offending agent. This is a case where a patient presented with symptoms of systemic lidocaine toxicity despite therapeutic dosage and an undetectable serum lidocaine concentration. A 47-year-old Caucasian male received a lumbar medial branch injection of lidocaine and presented with symptoms of perioral numbness, seizure-like activity, and confusion. The patient had a workup, including a CT head without acute findings and an undetectable serum lidocaine level. Due to symptoms consistent with systemic local anesthetic toxicity, intravenous lipid emulsion (ILE) was administered with resolution and without recurrence of symptoms. There should be a low threshold of suspicion for local anesthetic systemic toxicity when patients have neurologic or cardiovascular symptoms following exposure. Toxicity may be present despite therapeutic dosages and low serum concentrations. ILE may be beneficial and should be considered.

## Introduction

Lidocaine is a commonly used local anesthetic used in many medical procedures. Although generally considered safe and well tolerated, administration of lidocaine, as well as other local anesthetics, can produce systemic toxicity. Typically, the degree of severity is directly proportional to the serum concentration of the offending agent and is more likely if amounts over therapeutic dosage are used [1]. This case report describes a patient with symptoms of local anesthetic toxicity following a lumbar medial branch block using a therapeutic dosage of lidocaine with undetectable serum lidocaine levels. Symptoms were successfully abated following intravenous lipid emulsion (ILE) administration, suggesting that toxicity may still be present despite therapeutic dosage and low serum concentration.

## Case presentation

A 47-year-old male presented by private vehicle to the emergency department with seizure-like activity after receiving a lumbar lidocaine medial branch block at an outpatient pain clinic for pain associated with previous spinal trauma. The patient had previously tolerated the same procedure a few weeks prior without complications. He had no significant medical comorbidities and no history of substance use. The patient’s wife reported that approximately 20 minutes after injections, while she was driving the patient back home from the clinic, the patient developed perioral numbness and dysarthria. The patient’s symptoms progressed rapidly, with an abrupt decline in mental status followed by seizure-like activity. Upon arrival at the ED, the patient remained confused. There were no motor or sensory deficits noted on the physical exam.

**Figure 1:**
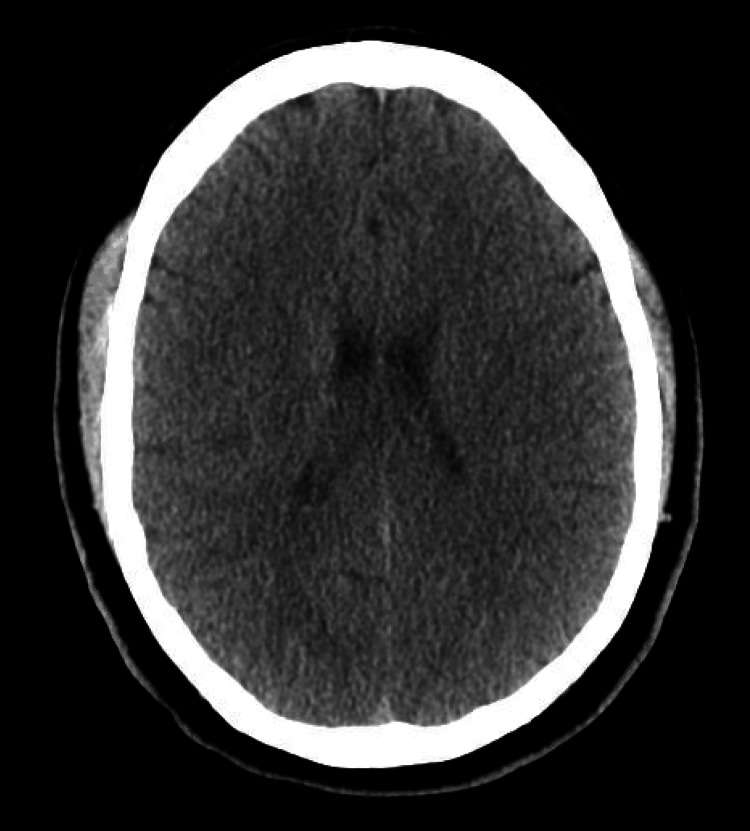
CT head without contrast Noncontrast axial images through the brain revealed no signs of acute intracranial hemorrhage, mass effect, or midline shift. Ventricular size was within normal limits for the patient’s age, and CSF spaces appeared relatively symmetric.

**Figure 2:**
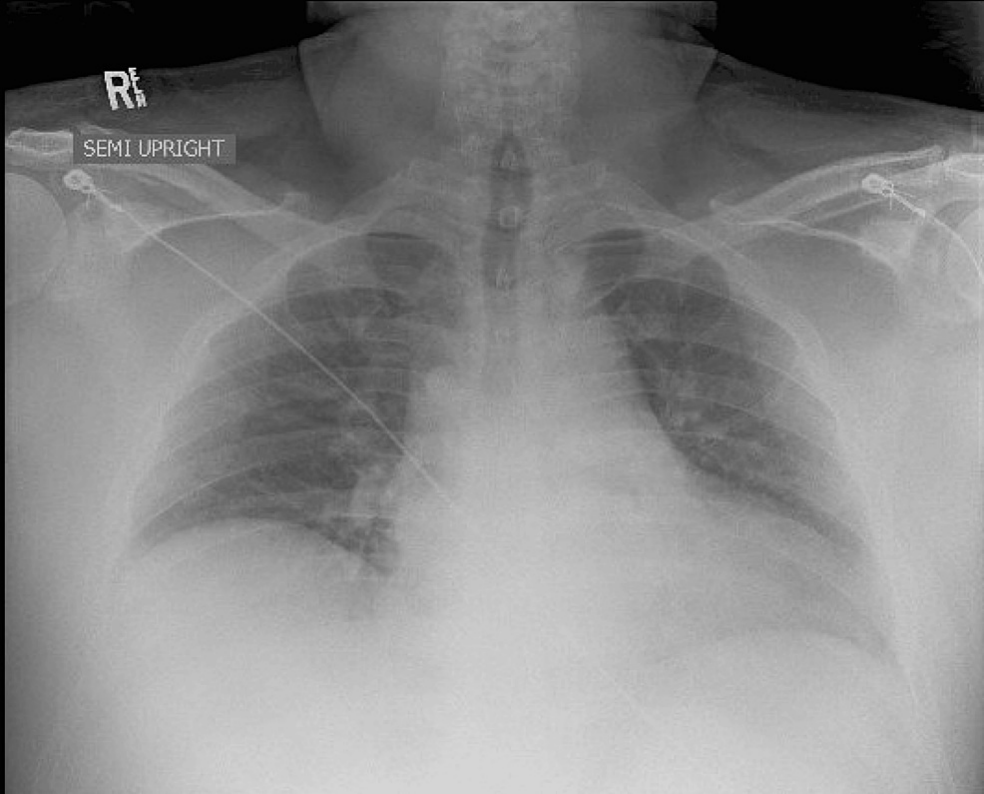
Chest X-ray The chest x-ray showed a normal configuration of the cardiomediastinal silhouette with no signs of acute consolidation. Sharp costophrenic angles were observed, and the pulmonary vasculature appeared unremarkable.

**Figure 3:**
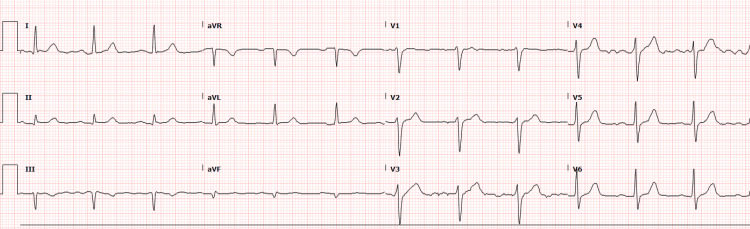
12-lead ECG Normal sinus rhythm; QRS duration: 109 ms; prolonged PR interval: PR >210 ms; inferior infarct, old: Q >35 mS, II III aVF

The patient had reportedly been administered 20 mL of 10 mg/mL lidocaine without epinephrine for skin numbing and 6 mL of 20 mg/mL lidocaine without epinephrine for injection, totaling 320 mg of lidocaine. Given the patient’s weight of 133.9 kg, which falls within accepted weight-based therapeutic dosing (<3 mg/kg), the expected dose would be 401.7 mg for this patient [2]. CT imaging of the head revealed no acute intracranial process (Figure 1), while the chest X-ray showed no acute intrathoracic process (Figure 2). Laboratory tests indicated no significant metabolic abnormalities or leukocytosis suggestive of an infectious process (Table 1). The EKG exhibited normal sinus rhythm with a slightly prolonged PR interval at 210 ms and evidence of an old inferior infarct (Figure 3). Upon arrival at the emergency department, the serum lidocaine level was less than 1 mcg/mL.

**Table 1:** Laboratory values ALT, alanine aminotransferase; AST, aspartate aminotransferase; BUN, blood urea nitrogen; CBC, complete blood count; GFR, glomerular filtration rate; Hgb, hemoglobin; INR, international normalized ratio; PCP, phencyclidine; THC, tetrahydrocannabinol; TSH, thyroid-stimulating hormone

Test name	Result	Reference range
Venous blood gas		
pH, venous	7.38	7.32-7.45
pCO2, venous	43 mmHg	38-54 mmHg
pO2, venous	62 mmHg	25-40 mmHg
HCO3-	24 mmol/L	22-28 mmol/L
O2 saturation, venous	90%	60-80%
Base excess	-0.3 mmol/L	-2-2 mmol/L
Barometric pressure	757 mmHg	752-767 mmHg
CBC with differential		
White cell count	6.3 × 10^3^/mcL	4.8-10.8 × 10^3^/mcL
Red cell count	5.18 × 10^6^/mcL	4.40-6.20 × 10^6^/mcL
Hemoglobin	15.7 g/dL	14.0-16.0 g/dL
Hematocrit	44.80%	41.0-53.0%
Mean cell volume	86.4 fL	80.0-100.0 fL
Mean cell Hgb	30.4 pg	27.0-32.0 pg
Mean cell Hgb concentration	35.1 g/dL	32.0-36.0 g/dL
Red cell distribution width	14.10%	12.0-15.0%
Platelet count	160 × 10^3^/mcL	140-450 × 10^3^/mcL
Mean platelet volume	9.2 fL	6.6-9.3 fL
Neutrophil %	62.10%	50.0-78.0%
Lymphocyte %	24.40%	20.0-35.0%
Monocyte %	10.20%	0.0-0.8%
Eosinophil %	2.80%	0.0-4.0%
Basophil %	0.50%	0.0-2.0%
Absolute neutrophil	3.9 × 10^3^/mcL	2.40-8.42 × 10^3^/mcL
Absolute lymphocyte	1.5 × 10^3^/mcL	0.96-3.78 × 10^3^/mcL
Absolute monocyte	0.6 × 10^3^/mcL	0.00-0.86 × 10^3^/mcL
Absolute eosinophil	0.2 × 10^3^/mcL	0.00-0.43 × 10^3^/mcL
Absolute basophil	0 × 10^3^/mcL	0.00-0.22 × 10^3^/mcL
Coagulation studies		
Activate partial thromboplastin time	32.4 seconds	25.1-36.5 seconds
Prothrombin time	12.5 seconds	9.4-12.5 seconds
INR	1.1	0.8-1.1
Comprehensive metabolic panel		
Sodium	140 mmol/L	136-145 mmol/L
Potassium	3.9 mmol/L	3.5-5.1 mmol/L
Chloride	106 mmol/L	98-107 mmol/L
CO2	26 mmol/L	21-32 mmol/L
Glucose, whole blood	109 mg/dL	74-106 mg/dL
Calcium, ionized	1.2 mmol/L	1.12-1.32 mmol/L
Magnesium level	2.1 mg/dL	1.8-2.4 mg/dL
Phosphorus level	3.5 mg/dL	2.5-4.9 mg/dL
BUN	17 mg/dL	7-25 mg/dL
Creatinine level	0.9 mg/dL	0.7-1.3 mg/dL
Estimated creatinine clearance	112.83 mL/min/BSA	77-160 mL/min/BSA
Estimated GFR	102 mL/min/1.73 m2	>60 mL/min/1.73 m2
Total protein	6.5 g/dL	6.4-8.2 g/dL
Albumin level	3.9 g/dL	3.4-5.0 g/dL
Total bilirubin	0.6 mg/dL	0.3-1.0 mg/dL
Alkaline phosphatase	63 unit/L	34-104 unit/L
ALT	98 unit/L	7-52 unit/L
AST	50 unit/L	13-39 unit/L
Direct bilirubin	0.14 mg/dL	0.03-0.18 mg/dl
Serum toxicology		
Lidocaine level	<1 mcg/mL	1.5-5.0 mcg/ml
Serum acetaminophen	<0.1 mcg/mL	10.0-30.0 mcg/mL
Serum ethanol	<10 mg/dl	0-50 mg/dL
Serum salicylate	<1.5 mg/dL	3.0-20.0 mg/dL
Urine drug screen		
Amphetamines	Negative	Negative
Barbiturates	Negative	Negative
Benzodiazepine	Negative	Negative
Opiates	Negative	Negative
Cocaine	Negative	Negative
THC	Negative	Negative
Fentanyl	Negative	Negative
Methadone	Negative	Negative
Ecstasy	Negative	Negative
PCP	Negative	Negative
Urinalysis		
Color	Yellow	N/A
Clarity	Clear	N/A
pH	5	5.0-7.5
Specific gravity	1.02	1.002-1.030
Protein	Negative	Negative
Glucose	Negative	Negative
Ketones	Negative	Negative
Bilirubin	Negative	Negative
Blood	Negative	Negative
Nitrite	Negative	Negative
Urobilinogen	<2 mg/dL	<2 mg/dL
Leukocyte esterase	Negative	Negative
Red cells	0/HPF	0-2/HPF
White cells	<1/HPF	0-5/HPF
Other studies		
Methemoglobin	1.20%	0-3%
Troponin-I HS	4 pg/mL	<20 pg/mL
TSH	2.299 mIU/L	0.450-5.330 mIU/L
Ammonia level	37 pg/dL	16-53 pg/dL
Lactic acid, whole blood	1.4 mmol/L	0.5-2.0 mmol/L

The patient experienced several witnessed episodes lasting several seconds of seizure-like activity during his ED course, characterized by minimal responsiveness with staring, nystagmus, and brief apneic episodes followed by post-ictal periods. He was administered 1 mg of IV lorazepam for seizure control. Following consultation with the Poison Center, it was decided to administer ILE due to the symptoms suggestive of lidocaine-associated CNS toxicity, despite therapeutic lidocaine dosage and an undetectable serum lidocaine level. A loading dose of 100 mL IV (0.75 mL/kg) of ILE was administered, followed by an infusion of 250 mL over 15 minutes (7.5 mL/kg/hr). Shortly after receiving treatment, the patient’s symptoms resolved completely.

**Figure 4:**
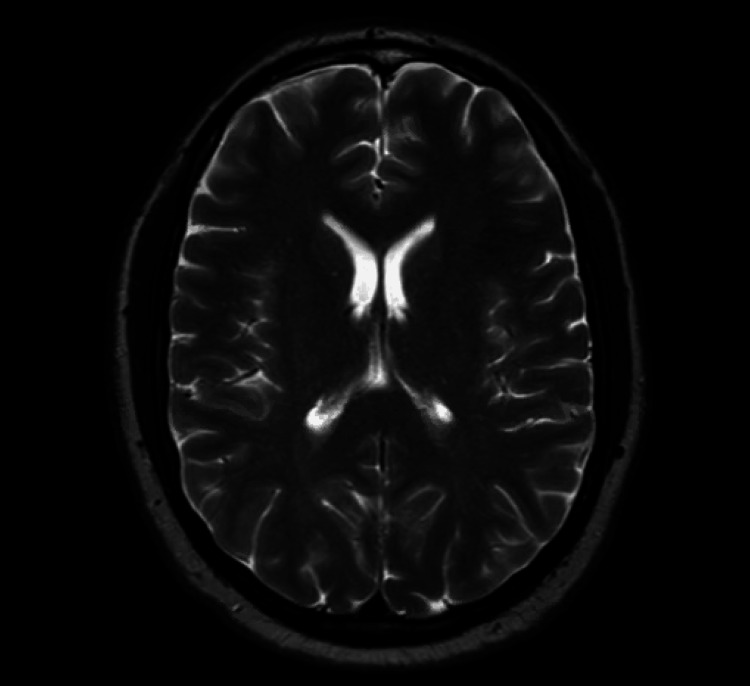
MRI brain without contrast No evidence of an acute stroke, hemorrhage, or mass was found. The ventricles appeared normal in size, with no midline shift observed. There was a sharp gray-white differentiation, and the appearance of the sulci and gyri was symmetric. The basilar cisterns were patent, and the paranasal sinuses were well aerated.

The patient was admitted to the ICU for further management of possible local anesthetic systemic toxicity (LAST). No repeated episodes of seizure-like activity were witnessed following ILE administration. MRI of the brain showed no acute intracranial disease, and electroencephalography did not show evidence of epileptiform activity (Figure 4). The patient’s presentation was ultimately attributed to lidocaine toxicity. Following an otherwise uncomplicated two-day hospital course, the patient was discharged home.

## Discussion

Pharmacology and pharmacokinetics of lidocaine

A member of the amide local anesthetics, lidocaine acts by reversibly binding to membrane-bound sodium channels in conducting tissues, blocking the initiation and propagation of action potentials. This creates an analgesic effect by inhibiting neuronal transmission along nerve fibers associated with pain [3]. Although this effect is generally associated with the peripheral nervous system, these types of conductive tissues are also present in the heart and brain.

**Figure 5:**
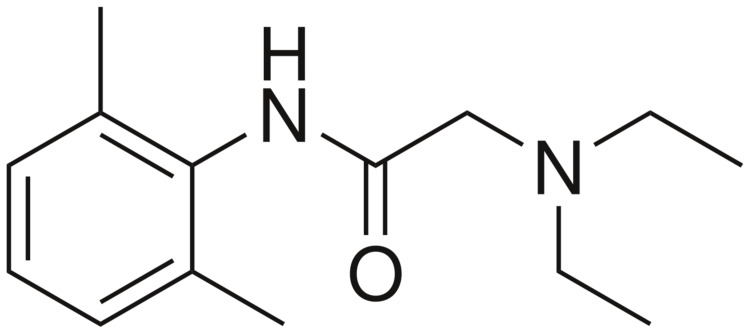
Chemical structure of lidocaine Lidocaine is a monocarboxylic acid amide that is formed from the condensation of N,N-diethylglycine with 2,6-dimethylaniline. Structurally, it consists of an aromatic group that binds to the target sodium channel and a lipophilic side chain.

The low molecular weight of local anesthetics is associated with rapid absorption and elimination. Because of their chemical structure, local anesthetics are lipophilic and have the propensity to readily cross cell membranes and the blood-brain barrier (Figure 5). Should molecules enter venous circulation, local anesthetics pass through pulmonary circulation, which acts as a saturable buffer [4]. Typically, seizures can be seen in intravascular boluses when pulmonary uptake of the substance exceeds 90%. The rapid injection can lead to high peak venous concentrations that exceed the capability of the lungs to buffer, leading to toxic arterial concentrations.

As an amino amide, lidocaine undergoes hepatic metabolization by CYP450 enzymes. Any factor that decreases hepatic blood flow or hinders hepatocellular function, such as age, medications, congestive heart failure, and liver disease, can increase the likelihood of toxic reactions [5,6].

Lidocaine central nervous system toxicity

Typically, systemic signs of local anesthetic toxicity (so-called LAST) correlate with the serum concentrations of the offending agent. At low levels (3-6 mcg/mL), symptoms of lightheadedness, perioral numbness, confusion, and auditory and visual disturbances can be seen. At higher concentrations of 5-9 mcg/mL, excitatory neurologic signs of shivering, tremors, and seizures may develop. Seizures are more likely to be seen at serum concentrations above 10 mcg/mL and may be accompanied by coma, apnea, and cardiovascular instability, with progression to respiratory and cardiac arrest in concentrations greater than 20 mcg/mL [7].

The development of CNS effects associated with lidocaine is also influenced by the rate of injection, drug interactions, acid-base status, and patient comorbidities. More rapid injections are associated with more profound toxicity. Metabolic and respiratory acidosis also increase CNS toxicity by decreasing protein binding and increasing CNS penetration. Hypercarbia may lower the seizure threshold by increasing cerebral blood flow and increasing delivery to the CNS, increasing the conversion of the lidocaine to the active cation, and decreasing protein binding [8-10].

CNS excitotoxicity is mediated by selective blockade of sodium channels in cerebral cortical inhibitory pathways of the amygdala, resulting in unopposed excitatory pathways and subsequent seizure phenomena. At higher concentrations, both excitatory and inhibitory pathways are blocked, contributing to CNS depression [11,12].

Diagnosis and treatment

LAST typically presents as increasingly worsening neurologic symptoms shortly after injection of a local anesthetic. Common neurological symptoms are perioral numbness, metallic taste, and auditory disturbances. The severity of symptoms typically parallels the serum concentration of the offending anesthetic [1]. When lidocaine toxicity is suspected, patients should be on continuous cardiac monitoring and should have an EKG to evaluate for cardiotoxicity. Laboratory evaluation should include a basic metabolic panel, blood gas analysis, and serum lidocaine levels, if available. Serum acetaminophen, ethanol, and salicylate levels, as well as urine drug screen, may help identify some co-ingestions, if present [7]. Lidocaine may also contribute to methemoglobinemia; blood gas analysis and co-oximetry are indicated in patients with hypoxemia or dyspnea [13].

In patients with symptoms of LAST, the administration of lidocaine must be discontinued. Patients with mental status changes or vital sign abnormalities may require ventilatory or vasopressor support. Therapeutic hyperventilation may help produce respiratory alkalosis, which in turn decreases CNS extraction of the drug, decreases extracellular potassium, normalizes pCO2, and decreases the affinity for and promotes dissociation of the agent from sodium channels [7].

Benzodiazepines are indicated as the first-line treatment for local anesthetic-induced seizures. If a patient should require endotracheal intubation, succinylcholine is not preferred as a paralytic agent as it is associated with hyperkalemia and dysrhythmias. For this reason, nondepolarizing neuromuscular blockade is preferentially recommended [7].

ILE is indicated in cardiac arrest associated with local anesthetic toxicity. The mechanism of action of ILE is not clearly understood. There are several proposed mechanisms, but the most accepted is the “lipid sink, sponge, conduit” mechanism. This theorizes that ILE essentially soaks up the offending agent and removes it from the area of toxicity. It is thought that ILE redistributes the compound to an area of high lipid content, hence the name “lipid conduit.” In rodent models, ILE decreased the distribution of bupivacaine in tissues of the brain and myocardium [14]. Although it has been debated whether it should be administered during early, milder signs of toxicity versus being reserved for cases of cardiovascular collapse, many authors agree that ILE should be administered when there are rapid CNS effects such as agitation, confusion, and seizures [15].

Neurotoxicity in low plasma levels of local anesthetics

There have been other cases of seizures after local anesthetic administration, despite low serum levels. One such case was an 18-year-old male who developed generalized tonic-clonic seizure phenomena after a brachial plexus block with ropivacaine. The patient’s serum ropivacaine level was 2.13 mg/L [16]. The arterial concentrations of ropivacaine associated with neurological symptoms are estimated to be 3.4-5.3 mg/L [17]. The patient’s seizures abated with midazolam. An EEG obtained two days following the event showed mild, localized, sharp waves at both temporal lobes, suggesting that the patient may have had underlying epilepsy [16].

In a similar case, a 48-year-old female developed confusion and generalized seizures after an epidural injection of ropivacaine. Seizure activity ceased after midazolam administration. Arterial blood gas showed acidosis with a pH of 7.181 and a base excess of -10.7, for which sodium bicarbonate was subsequently given. The arterial plasma ropivacaine level was 1.5 mg/L [18].

Another possible explanation for the observed neurological symptoms in this case would be an inadvertent subdural injection of lidocaine. A case report documented flaccid paralysis and irregular respirations in a 40-year-old female immediately after a C6 nerve root block that self-resolved in 20 minutes. CT imaging showed air gases, suggesting a subdural air infusion [19]. Other case reports have documented intra-arterial, subdural, and intrathecal injections [20]. In the presented case, however, there were no findings on imaging suggesting subdural injection.

## Conclusions

This was a case of lidocaine-associated neurotoxicity despite therapeutic dosage and an undetectable serum lidocaine level that was successfully treated with ILE. In this case, the patient’s symptomology was consistent with LAST. Although other cases of LAST despite negative serum levels of other anesthetic agents have been documented, few specifically related to lidocaine have been reported. Clinicians should have a high level of suspicion for LAST if a patient presents with neurological symptoms or cardiovascular instability following the administration of local anesthetics. In such a case, ILE administration should be considered following a discussion with a medical toxicologist.
